# The relationship between tumour infiltrating lymphocytes, Epstein–Barr virus and *Helicobacter pylori* infection in gastric cancer

**DOI:** 10.3332/ecancer.2022.1362

**Published:** 2022-03-01

**Authors:** Carlos Castañeda, Miluska Castillo, Luis Bernabe, Nancy Suarez, Matteo Fassan, Joselyn Sanchez, Katherine Tello, Raul Alatrista, Ivan Chavez, Eloy Ruiz, Yaqueline Bazan, Fernando Barreda, Daniel Valdivia, Wei Meng, Arnab Chakravarti, Juvenal Sanchez, Luis Taxa, Paola Montenegro

**Affiliations:** 1 Facultad de Ciencias de la Salud, Universidad Cientifica del Sur, Lima 15067, Peru; 2 Departamento de Oncologia Medica, Instituto Nacional de Enfermedades Neoplasicas, Lima 15038, Peru; 3 Departamento de Investigación, Instituto Nacional de Enfermedades Neoplasicas, Lima 15038, Peru; 4 Department of Medicine-DIMED, Surgical Pathology Unit, University of Padua, Padua 35122, Italy; 5 Veneto Institute of Oncology, IOV-IRCCS, Padua 35128, Italy; 6 Departamento de Cirugía en Abdomen, Instituto Nacional de Enfermedades Neoplasicas, Lima 15038, Peru; 7 Departamento de Especialidades Médicas, Instituto Nacional de Enfermedades Neoplasicas, Lima 15038, Peru; 8 Department of Radiation Oncology, The Ohio State University Wexner Medical Center, Columbus, OH 43210, USA; 9 Departamento de Patologia, Instituto Nacional de Enfermedades Neoplasicas, Lima 15038, Peru; a https://orcid.org/0000-0001-6200-0856; b https://orcid.org/0000-0002-0111-3176; c https://orcid.org/0000-0003-1896-7060; d https://orcid.org/0000-0001-5955-3919; e https://orcid.org/0000-0001-6515-5482; f https://orcid.org/0000-0002-6764-4180; g https://orcid.org/0000-0002-4981-3411; h https://orcid.org/0000-0002-3431-3262; i https://orcid.org/0000-0002-4644-0074; j https://orcid.org/0000-0002-7337-2396; k https://orcid.org/0000-0002-7923-6299; l https://orcid.org/0000-0002-5917-6452; m https://orcid.org/0000-0002-9825-8573

**Keywords:** Epstein–Barr virus, Helicobacter pylori, lymphocytes, in situ hybridisation

## Abstract

**Objective:**

Epstein–Barr virus (EBV) and *Helicobacter pylori* (HP) infections have been extensively recognised as gastric cancer (GC) triggers, and recent publications suggest they could behave as predictive markers for immune-modulating therapies. Tumour-infiltrating lymphocytes (TILs) have also been identified as a predictive biomarker for immunotherapy in different malignancies. This study aimed to investigate the association between EBV and HP infection with TIL levels in GC.

**Methods:**

TIL evaluation in haematoxylin-eosin was performed by a pathologist and density of CD3, CD8 and CD163 positive (immunohistochemistry staining) immune cells was calculated with the use of digital pathology software. EBV infection was detected by *in situ* hybridisation (ISH) and by quantitative polymerase chain reaction (qPCR). Methylation status of EBV-related genes was detected by PCR and a methylome analysis was performed by the Illumina Infinium MethylationEPIC BeadChip. HP status was detected by qPCR.

**Results:**

We included 98 resected GC Peruvian cases in our evaluation. Median TIL percentage was 30. The proportion of EBV+ detected by ISH was 24.1%, of EBV+ detected by qPCR was 41.8%, while 70% showed methylation of EBV-related genes, and 58.21% of cases were HP+. Younger age (*p* = 0.024), early stages (*p* = 0.001), HP+ (*p* = 0.036) and low CD8 density (*p* = 0.046) were associated with longer overall survival (OS). High TIL level was associated with intestinal subtype (*p* < 0.001), with grade 2 (*p* < 0.001), with EBV qPCR+ (*p* = 0.001), and with methylation of EBV-related genes (*p* = 0.007). Cases with high TIL level and cases that are EBV positive share eight genes with similarly methylated status in the metabolomic analysis. High CD8 density was associated with EBV PCR+ (*p* = 0.012) and HP− (0.005).

**Conclusion:**

Lower CD8 density and HP+ predict longer OS. High TIL level is associated with EBV+ and methylation of EBV-related genes, while lower CD8 density is associated with HP+ GC.

## Introduction

Gastric cancer (GC) represents the fourth most common type of cancer and the second leading cause of cancer death worldwide. The incidence of GC varies up to 10-fold by geographic region, suggesting that genetic or environmental factors influence carcinogenesis and clinicopathological features. In Peru, GC is the second in males and the third most frequent malignancy in women [1]. Epstein–Barr virus (EBV) is uniformly present in gastric adenocarcinoma subtype with extensive lymphocyte infiltration [2, 3] and is associated with cytosine-guanine (CpG) dinucleotide hypermethylated pattern and longer survival [4, 5]. Recent studies indicate that EBV presence could also predict efficacy of checkpoint immune inhibitors in GC [6].

Persistent infection with *Helicobacter pylori* (HP) induces chronic inflammation and gastric carcinogenesis. HP prevalence in adults living in developing Latin American countries is 70% to 80%, and its presence in GC has been suggested to be associated with a better prognosis and response to checkpoint immune inhibitors [7, 8].

Some reports have shown that a high level of tumour-infiltrating lymphocytes (TILs) is related to both longer survival in GC [9] and a higher response to checkpoint inhibitors. However, methodologies for evaluating TILs have been diverse, and only a few studies have evaluated the relationship between TIL levels and clinicopathological features [10–13]. Recently, the International Immuno-Oncology Biomarkers Working Group (IBWG) has proposed a standardised methodology to evaluate TIL in different malignancies including gastric carcinoma [14]. Furthermore, some recent reports have described that density of immune cell subpopulations belonging to TIL like CD3 positive (T-cell) lymphocytes, CD8 positive (cytotoxic T-cell) lymphocytes and CD163 positive (pro-tumour M2) macrophages could be responsive to the mentioned associations [13, 15].

We developed an exploratory study to evaluate the relation between the TIL level based on IBWG methodology [6, 14] and the infection of HP and EBV in a cohort of EBV enriched GC population. Additionally, density of CD3, CD8-and CD163positive immune cell subpopulations and their relationship with HP and EBV infections were calculated. Evaluation of EBV infection included the analysis of methylation status of EBV-related genes.

## Materials and methods

### Subjects

The study included histology confirmed gastric adenocarcinomas from patients who underwent surgery at the Instituto Nacional de Enfermedades Neoplasicas (Lima–Peru) from 2015 to 2018. All the patients were selected by convenience sampling. Cases were selected among those with the highest EBV gene counts by polymerase chain reaction (PCR) (previously selected [1] with counts that reached up to 166,210.5 copies/μL) in order to obtain a group with high rates of positive *in situ* hybridisation (ISH) evaluations for EBV. All included cases had ISH status for EBV and/ or methylation evaluation of EBV-related genes.

This single-centre retro-prospective cohort research was presented and approved by the Research and Ethics committee (Protocol Number #050-2015-CIE/INEN), and the patients were invited to read and sign the informed consent.

### Tumour specimens

Every tumour sample was collected and stored at −80°C until use at the Institute Biobanking for quantitative PCR (qPCR) for EBV, as well as paired Formalin-Fixed Paraffin-Embedded (FFPE) samples saved at pathology archive. Tissue microarrays (TMAs) were constructed from tumour cores (6.0 mm diameter) through the invasive areas of each specimen from the selected FFPE blocks. Serial 4-μm sections were prepared and used for immunohistochemistry (IHC) staining [6].

### Immunohistochemistry (IHC)

Sections from paraffin samples were rehydrated in phosphate-buffered saline (PBS), and antigen retrieval was performed by immersing in 0.1% trypsin solution in PBS at 37°C for 5–10 minutes or by microwave heating for 5 minutes × 4 (total, 20 minutes) in buffer solution. The sections were treated for 45 minutes with 10% normal goat serum or normal horse serum in PBS. The primary antihuman antibodies used for IHC were CD3 (IS503, Dako, Glostrup, Denmark), CD8 (clone C8/144B, IS623, Dako, Glostrup, Denmark) and CD163 (clone EP324, Master Diagnostica, Granada, España). The sections were incubated further in alkaline phosphatase–streptavidin (Vector Laboratories, Burlingame, CA; 1:1,000 dilution) for 30 minutes at room temperature, reacted with Fast-Red Substrate System (Dakopatts) or with Dako® Fuchsin + Substrate-Chromogen. Background staining was performed with Mayer’s haematoxylin solution, and sections were then dehydrated through ascending alcohols to xylene and mounted on slides.

### Measurement of TILs

**Figure 1.**
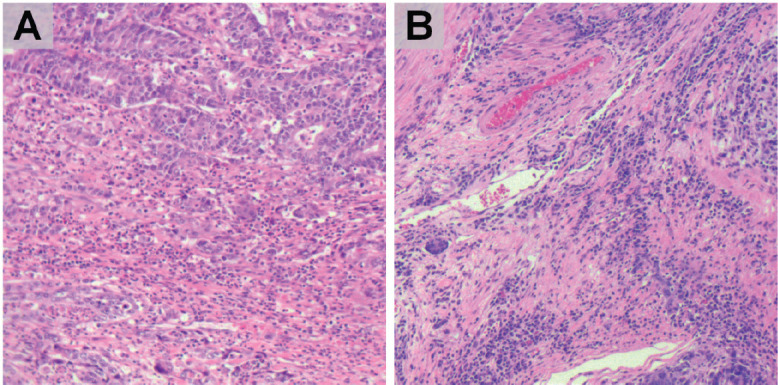
HE staining of GC. Representative slides of stromal compartment with high (a, b) level of tumour infiltrating lymphocytes. (magnification: ×200).

**Figure 2.**
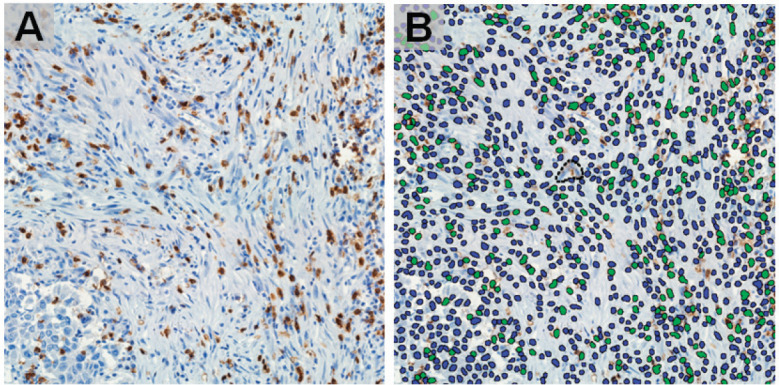
Identification of CD8 in IHC staining images (a) by machine learning-based image processing (b) showing positive (green) and negative (blue) cells, (magnification: ×400).

FFPE tissue samples were retrieved and haematoxylin-eosin stained slides were obtained. The IBWG method for TIL assessment in the stromal compartment was applied by one pathologist (J Sanchez) (Figure 1). The densities of T lymphocytes were assessed following our previous report [6]. Immunostained slides were digitally scanned using a BX63 Olympus scanner (Olympus, Tokyo, Japan) at 20× magnification. Digital images were viewed with Visiopharm Integrator System software version 6.6.1.2572 (Visiopharm, Hørsholm, Denmark). Density of CD3, CD8 and CD163-positive cells were calculated, in cases with enough stained tissue, by VisionPharm software by counting the number of positive cells for staining/total number of cells in five high power fields located in the stromal compartment (M Castillo and LA Bernabe) under supervision by a pathologist (J Sanchez) [6] (Figure 2).

### EBV ISH

Chromogenic ISH for EBV-encoded RNA (EBER) was performed in FFPE tissue samples with ﬂuorescein-labelled oligonucleotide probes (EBER probe, Ventana) with enzymatic digestion (ISH protease 3, Ventana) and an iViewBlue detection kit (Ventana) with use of the BenchMark ULTRA staining system (K Tello).

### Evaluation of the EBV gene and HP gene expression

The detection of EBV gene expression was performed in a region of *BNRF1*, and HP genes by the *hspA* and *UreA* genes in DNA from frozen samples as targets by qPCR in the LightCycler 96 Instrument Thermal Cycler (Roche, Mannheim, Germany) as described in our previous publication [1]. Values were considered as positive when ≥10 copies/μL was detected (N Suarez).

### Evaluation of methylation in genes related to EBV

Genomic DNA was isolated from GC frozen samples using the classical method of phenol/chloroform/isoamylalcohol and proteinase K. The bisulfite treatment was performed as previously described with the EpiTect Bisulfite (Qiagen, Germany) kit and the thermal cycler Mastercycler nexus gradient (Eppendorf, Germany). The methylation status of six gene promoters (*RASSF1*, *CDKN2A*,* MGMT*, *GSTP1*, *HOXA10* and *TP73*) were analysed in the LightCycler® 96 Instrument (ROCHE®) thermal cycler, and the results were interpreted regarding the threshold cycle presence in the software LightCycler® 96 System Version 2.0 [5]. Primer sequences were forward: 5′ TGGAGTTTTCGGTTGATTGGTT 3′ and reverse: 5′ AACAACGCCCGCACCTCCT 3′ for *CDNK2A*; forward: 5′ ATCGGAAGTGCGTTATTTCGTG 3′ and reverse: 5′ TTCCGTCTCTCGACTCGAAACT 3′ for *HOXA10*; forward: 5′ GGGTCGGGTAGTTCGTTTTG 3′ and reverse: 5′ CGATTTCGCTACGTCCCCT 3′ for *TP73*; forward: 5′ ATTGAGTTGCGGGAGTTGGT 3′ and reverse: 5′ ACACGCTCCAACCGAATACG 3′ for *RASSF1A*; forward: 5′ GTCGGCGTCGTGATTTAGTATTG 3′ and reverse: 5′ AAACTACGACGACGAAACTCCAA 3′ for *GSTP1*; forward: 5′ GCGTTTCGACGTTCGTAGGT 3′ and reverse: 5′ CACTCTTCCGAAAACGAAACG 3′ for *MGMT*.

DNA methylation profiling was performed using the Illumina Infinium MethylationEPIC BeadChip that features over 850,000 CpGs in enhancer regions, gene bodies, promoters, and CpG islands according to the manufacturer’s instructions, in FFPE tumour tissues of a subset of 24 GC cases in the Molecular Genomics Core lab, USC Norris Comprehensive Cancer Center (Los Angeles, CA). The methylation values for the individual CpG sites were obtained as *β* values. The *β*-value generated for each CpG locus reflected a measure of the percentage of the methylated (*β* = 1) and unmethylated probes (*β* = 0). The *β*-values of each probe are continuous variables that are calculated by dividing the intensity of the methylated probe by the combined methylated and unmethylated probe intensities, and the resultant values range from 0 to 1.

### Statistical analysis

Comparisons of categorical variables were done by a Chi-square test or Fisher’s exact test as appropriate. Correlation between density of CD3, CD8 and CD163-positive cells were calculated by intraclass correlation coefficient (ICC). Overall survival (OS) was defined as the time from the date of surgery until death from any cause or last vital status obtained from patient file or from National Registry of Identification and Marital Status webpage (www.reniec.gob.pe). The follow-up for vital status was completed in September 2021. Survival rates were estimated by the Kaplan–Meier method. All tests were two-sided, and differences were considered to be significant when *p* < 0.05. Statistical analyses were performed with IBM SPSS Statistics version 21 (IBM, Armonk, NY). Genome-wide DNA methylation was analysed, identifying differentially methylated regions (DMR) with respect to TIL level (with a cutoff of 30%) and ISH EBER status (positive or negative) through Illumina Infinium MethylationEPIC BeadChip. The raw intensity data were imported into R (3.6.0, https://cran.r-projecto.org/) and then analysed with the minfi package for data preprocessing normalisation and comparison between groups. Statistical analyses of the data were performed in RStudio (1.2.1335) (http://www.rstudio.com/) using an R environment (3.6.0) (https://www.R-project.org).

## Results

### Clinicopathological features

In the whole series of 98 cases, the median age was 68 years and 41.8% were women. Clinicopathological features are described in Table 1. HP was detected in 56.1%. Median TIL percentage was 30 (interquartile range (IQR) = 10%–60%). Median densities of CD3 positive cells, CD8 (T lymphocytes) and CD163 positive cells were 21.07% (IQR = 10%–60%%), 10.48% (IQR = 5.77%–20.04%) and 11.17% (IQR = 5.68%–17.29%), respectively. Density of CD3 positive cells had close correlation with CD8 (ICC = 0.834 (95% CI: 0.725 to 0.899, *p* < 0.001)). Density of CD3 positive cells (ICC = 0.241 (95% CI: −0.33 to 0.567, *p* = 0.167)) had poor and CD8 (ICC = 0.551 (95% CI: 0.183 to 0.753, *p* = 0.005)) had a high direct correlation with CD163.

**Figure 3.**
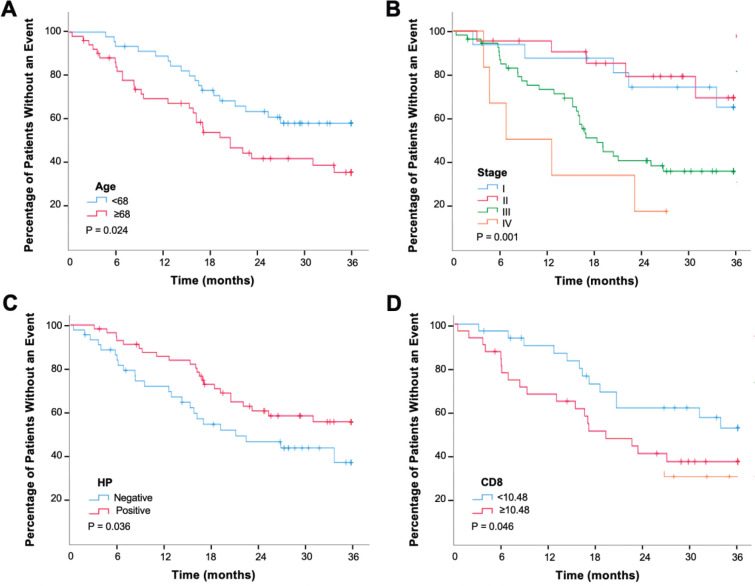
Kaplan–Meier curve for OS by age (a), stage (b), HP status (c) and CD8 positive T cell density (d).

Median follow up was 21.7 months and 47 patients (48%) were alive at 3 years. Older age (*p* = 0.024), presence of lymphovascular invasion (*p* = 0.007), advanced pathological stage (*p* = 0.001), recurrence (*p* = 0.002), HP absence (*p* = 0.036) and high CD8 density (*p* = 0.046) were associated with shorter OS (Figure 3). The level of TIL (*p* = 0.594), CD3 density (*p* = 0.241) and CD163 density (*p* = 0.152) were not associated with survival (Table 1).

### Determination of EBV status through ISH, qPCR and methylation

Status for EBV was positive using EBER-ISH method in 19 of the 79 (24.1%) evaluated cases and was positive using qPCR method in 41 of 98 (41.8%) cases. There was no significant relationship between EBER ISH and qPCR detection (kappa index = 0.012, *p* = 0.864). There was no significant association between OS and EBER ISH (*p* = 0.345) or qPCR (*p* = 0.809).

An upregulated methylation status in at least one gene was found in 55 of the 78 (70%) evaluated cases (Table 1). There was methylation of *RASSF1*, *CDKN2A*, *MGMT*, *GSTP1*, *HOXA10* and *TP73* in 37.2%, 48.7%, 34.6%, 11.5%, 15.4% and 10.3%, respectively. There was a significant association between methylation status and presence of EBV infection evaluated through qPCR (kappa index = 0.328, *p* = 0.003) or ISH (kappa index = 0.158, *p* = 0.025).

The methylome analysis using Infinium MethylationEPIC BeadChip in 24 tumour samples found that DMR cutoff >30% with <0.05 *p*-value regarding EBER ISH status identified 88 DMR-related genes (from 116 DMRs).

### Association between TILs and clinicopathological features in gastric tissues

High TIL level was associated with intestinal subtype (*p* < 0.001) and well/ moderately differentiated grade (*p* < 0.001). CD3 density was not associated with HP (*p* = 0.531) nor other variables (*p* > 0.01). High CD8 density was associated with absence of HP infection. High CD163 density was associated with male gender (*p* = 0.02), diffuse subtype (0.040) and stage III (*p* = 0.02) (Table 2).

### Association between TILs and EBER-ISH status, Epstein-Barr gene expression or methylation signature

High TIL level and high CD8 density were associated with positive EBV status through qPCR (*p* = 0.001 and *p* = 0.012, respectively) and a trend with EBER ISH status (*p* = 0.098 and 0.077, respectively) (Table 1). High TIL level was also associated with the methylation of at least one of the 6 EBV-related genes (*p* = 0.007) and methylation of *RASSF1A* (*p* = 0.011). Additionally, high density of CD8-positive cells was associated with the methylation status of *RASSF1A* (*p* = 0.048) and *CDKN2A* (*p* = 0.025) (Table 2). CD3 density wasnot associated with EBER ISH (*p* = 0.124) or with other EBV evaluation (*p* > 0.05).

The methylome analysis using Infinium MethylationEPIC BeadChip found that a DMR cutoff>30% with a <0.05 *p*-value identified 32 DMRs related genes (from 146 DMR) associated with >30% TIL. Furthermore, eight genes were shared among EBER ISH positive status and TIL>30%: *HEATR4* (HEAT repeat containing 4) (high), *RGMA* (repulsive guidance molecule BMP co-receptor a) (high), *MTRNR2L1* (MT-RNR2like 1) (low), *SH2D4A* (SH2 domain containing 4A) (low), *SORCS3* (sortilin related VPS10 domain containing receptor 3) (low), *TBC1D14* (TBC1domain family member 14) (low), *TMEM260* (transmembrane protein 260) (low) and *TNNT3* (troponin T3, fast skeletal type) (low).

## Discussion

We found a remarkable association between TIL level and EBV infection. High TIL level and density of CD8 T lymphocytes were significantly associated with positive EBV status and methylation of EBV-related genes.

Methylation patterns have been extensively described in EBV-positive GC [5], and our finding that the TIL level was also associated with methylation in at least one of six evaluated cancer genes suggests that methylation process could mediate the immune activity against EBV-infected tumours. This explanation is also supported by our finding that altered methylation status of eight genes is similar in those tumours with high TIL level and in those with EBER ISH positive status in the genome-wide DNA methylation analyses. *SORCS3* is one of the genes with altered methylation and has been previously described to be related to gastrointestinal tumour progression [16, 17]. Hanahan [20] concluded that nonmutational epigenetic reprogramming is a characteristic process that facilitates the acquisition of hallmark capabilities, and recent studies find that methylation of tumour genes can mediate ability of the immune system to detect GC cells [18, 19]. The hypothesis that the association between EBV infection and high TIL level (and high CD8 density) is mediated by the methylated pattern deserves further research because it could lead to the identification of tumour biomarkers that predict activity of immunotherapy in GC.

A significant association between high density of CD8-positive lymphocytes and shorter survival was also found in our series. This effect could be related to the previously described direct correlation between CD8 T lymphocytes and the level of pro-tumour PD-L1 and FOXP3 positive T lymphocytes [10–13, 21]. High density of CD163 positive cells (M2 macrophages) was associated with aggressive features like diffuse subtype and stage III disease and had a trend to shorter survival (39% versus 56% OS at 3 years, *p* = 0.24). This protumour effect has been previously described [22, 23] and our finding that it was directly correlated with CD8 T cell density could also add to the negative survival impact of CD8 density.

HP infection was also significantly associated with longer survival in our series, a relationship that has previously been described by other groups [7]. Furthermore, our finding that the good prognostic feature of a low density of CD8-positive lymphocytes is associated with the infection, suggests that an effective immune response against GC could explain the better prognosis of HP+ tumours.

Recent publications suggest that the tumour response to immunotherapy could be reduced in patients with HP infection [8]. This fact could be explained by our finding of reduced tumour infiltration by CD8 positive T cells.

A limitation of our exploratory research is the small size of our series that makes our results need to be validated in larger-size studies. The analysed tumour sample volume was small since we used TMAs; however, the evaluated area of invasive tumour was selected by a pathologist, and most samples obtained in real-world are similarly small as they are obtained from gastroscopies.

## Conclusion

In conclusion, we found that high level of TIL in GC was associated with EBV infection, and this association could be mediated by the methylation status. HP infection is associated with longer survival, and this association could be mediated by lower CD8 T cell infiltration.

## Conflicts of interest

The author(s) declare that they have no conflict of interest.

## Funding

Universidad Cientifica del Sur, Consejo Nacional de Ciencia, Tecnologia e Innovacion Tecnologica (under projects #198-2015-FONDECYT and #196-2015-FONDECYT), and INNOVATEPERU (under project #317-PNICP-EC-2014).

## Figures and Tables

**Table 1.** Clinicopathological features.

Features	*n*	%	OS at 3 years (%)	*p* value
Age (Median) (IQR = 17)				0.024^*^
<68	46	46.9	60.9	
≥68	52	53.1	44.2	
Gender				0.788^*^
Male	57	58.2	52.6	
Female	41	41.8	51.2	
Lauren subtype				0.507^*^
Intestinal	57	58.2	49.1	
Diffuse	28	28.6	53.6	
Mixed	13	13.3	61.5	
Histological grade^a^				0.301^*^
1	14	14.3	64.3	
2	33	33.7	45.5	
3-4	51	52.0	52.9	
Lymphovascular invasion				0.007^*^
No	34	34.7	67.6	
Yes	64	65.3	43.8	
Perineural invasion				0.167^*^
No	41	41.8	53.7	
Yes	57	58.2	50.9	
Location				0.183^*^
Antrum	54	55.1	55.6	
No-Antrum	44	44.9	47.7	
Resection				0.032^**^
Subtotal	57	58.2	56.1	
Total	41	41.8	36.6	
Pathological stage				0.001^*^
I	16	16.3	68.8	
II	22	22.4	77.3	
III	54	55.1	40.7	
IV	6	6.1	16.7	
Recurrence (*n* = 98)				0.002^**^
No	73	74.5	58.9	
Yes	25	25.5	16.0	
HP				0.036^*^
Negative	43	43.9	37.2	
Positive	55	56.1	56.4	
TIL stromal (Median)				0.594^**^
<30	48	49.0	52.1	
≥30	50	51.0	52.0	
CD3 density (Median) (*n* = 71)				0.241^*^
<21.07	35	49.3	54.3	
≥21.07	36	50.7	44.4	
CD8 density (Median) (*n* = 64)				0.046^*^
<10.48	32	50.0	59.4	
≥10.48	32	50.0	37.5	
CD8/CD3 ratio (*n* = 63)				0.361^**^
Low	31	49.2	51.6	
High	32	50.8	43.8	
CD163 density (Median) (*n* = 63)				
<11.17	32	49.2	56.3	0.152^*^
≥11.17	33	50.8	39.4	
EBER-ISH (*n* = 79)				0.345^*^
Negative	60	75.9	50.0	
Positive	19	24.1	42.1	
EBV (qPCR) (*n* = 98)				0.809^*^
Negative	57	58.2	54.4	
Positive	41	41.8	48.8	
Methylated EBV-related genes^b^ (*n* = 78)				0.494^*^
No	23	29.5	47.8	
Yes	55	70.5	52.7	

OS: Overall survival; TIL: Tumour-infiltrating lymphocytes; EBV: Epstein–Barr virus

a 1: Well differentiated; 2: Moderately differentiated; 3–4: Poorly differentiated and undifferentiated

b At least one gene

*p* value < 0.05.

* Breslow;

** Log Rank

**Table 2.** Evaluation of relationship among tumour-infiltrating-lymphocytes and clinicopathological features.

Features	TIL <30% (*n* = 48)	TIL ≥ 30% (*n* = 50)	*p*	CD8 <10.48	CD8 ≥10.48	*p*	CD163 <11.17	CD163 ≥11.17	*p*
Age (Median)			0.070^*^			0.8^*^			0.256^*^
<68	27 (58.7)	19 (41.3)		14 (51.9)	13 (48.1)		12 (41.4)	17 (58.6)	
≥68	21 (40.4)	31 (59.6)		18 (48.6)	19 (51.4)		20 (55.6)	16 (44.4)	
Gender			0.973^*^			0.313^*^			0.035*
Male	28 (49.1)	29 (50.9)		16 (44.4)	20 (55.6)		19 (63.3)	11 (36.7)	
Female	20 (48.8)	21 (51.2)		16 (57.1)	12 (42.9)		13 (37.1)	22 (62.9)	
Lauren subtype			<0.001^*^			0.955^**^			0.040^*^
Intestinal	17 (29.8)	40 (70.2)		21 (51.2)	20 (48.8)		23 (60.5)	15 (39.5)	
Diffuse	24 (85.7)	4 (14.3)		7 (46.7)	8 (53.3)		4 (23.5)	13 (76.5)	
Mixed	7 (53.8)	6 (46.2)		4 (50.0)	4 (50.0)		5 (50.0)	5 (50.0)	
Histological grade^a^			<0.001^*^			0.165^*^			0.081^*^
1	8 (57.1)	6 (42.9)		8 (72.7)	3 (27.3)		9 (75.0)	3 (25.0)	
2	6 (18.2)	27 (81.8)		13 (52.0)	12 (48.0)		11 (52.4)	10 (47.6)	
3–4	34 (66.7)	17 (33.3)		11 (39.3)	17 (60.7)		12 (37.5)	20 (62.5)	
Lymphovascular invasion			0.065^*^			0.171^*^			0.170^*^
No	21 (61.8)	13 (38.2)		12 (63.2)	7 (36.8)		15 (60.0)	10 (40.0)	
Yes	27 (42.2)	37 (57.8)		20 (44.4)	25 (55.6)		17 (42.5)	23 (57.5)	
Perineural invasion			0.658^*^			0.611^*^			0.531^*^
No	19 (46.3)	22 (53.7)		14 (53.8)	12 (46.2)		14 (45.2)	17 (54.8)	
Yes	29 (50.9)	28 (49.1)		18 (47.4)	20 (52.6)		18 (52.9)	16 (47.1)	
Location			0.149^*^			0.611^*^			0.105^*^
Antrum	30 (55.6)	24 (44.4)		20 (52.6)	18 (47.4)		20 (58.8)	14 (41.2)	
No-antrum	18 (40.9)	26 (59.1)		12 (46.2)	14 (53.8)		12 (38.7)	19 (61.3)	
Pathological stage			0.086^*^			0.095^**^			0.029^**^
I	11 (68.8)	5 (31.3)		6 (60.0)	4 (40.0)		7 (58.3)	5 (41.7)	
II	8 (36.4)	14 (63.6)		11 (68.8)	5 (31.3)		10 (76.9)	3 (23.1)	
III	28 (51.9)	26 (48.1)		15 (42.9)	20 (57.1)		11 (32.3)	23 (67.6)	
IV	1 (16.7)	5 (83.3)		0 (0.0)	3 (100.0)		4 (66.7)	2 (33.3)	
Recurrence			0.564^*^			0.095^*^			0.181^*^
No	37 (50.7)	36 (49.3)		26 (56.5)	20 (43.5)		26 (54.2)	22 (45.8)	
Yes	11 (44.0)	14 (56.0)		6 (33.3)	12 (66.7)		6 (35.3)	11 (64.7)	
HP (PCR)			0.213^*^			0.005^*^			0.702^*^
Negative	18 (41.9)	25 (58.1)		8 (29.6)	19 (70.4)		14 (46.7)	16 (53.3)	
Positive	30 (54.5)	25 (45.5)		24 (64.9)	13 (35.1)		18 (51.4)	17 (48.6)	
EBER-ISH			0.098^*^			0.077^**^			0.509^*^
Negative	32 (53.3)	28 (46.7)		19 (51.4)	18 (48.6)		19 (48.7)	20 (51.3)	
Positive	6 (31.6)	13 (68.4)		3 (23.1)	10 (76.9)		4 (33.3)	8 (66.7)	
EBV (qPCR)			0.001^*^			0.012^*^			0.914^*^
Negative	28 (68.3)	13 (31.7)		20 (66.7)	10 (33.3)		14 (50.0)	14 (50.0)	
Positive	20 (35.1)	37 (64.9)		12 (35.3)	22 (64.7)		18 (48.6)	19 (51.4)	
Methylated EBV- related genes			0.007^*^			0.174^*^			0.928^*^
No	16 (69.6)	7 (30.4)		10 (66.7)	5 (33.3)		8 (50.0)	8 (50.0)	
Yes	20 (36.4)	35 (63.6)		16 (45.7)	19 (64.7)		19 (51.4)	18 (48.6)	
*RASSF1* ^c^			0.011^*^			0.048^*^			0.915^*^
Negative	28 (57.1)	21 (42.9)		20 (62.5)	12 (37.5)		17 (51.5)	16 (48.5)	
Positive	8 (27.6)	21 (72.4)		6 (33.3)	12 (66.7)		10 (50.0)	10 (50.0)	
*CDKN2A* (*p* 16)^c^			0.484^*^			0.025^*^			0.218^*^
Negative	20 (50.0)	20 (50.0)		18 (66.7)	9 (33.3)		18 (58.1)	13 (41.9)	
Positive	16 (42.1)	22 (57.9)		8 (34.8)	15 (65.2)		9 (40.9)	13 (59.1)	
*MGMT* ^c^			0.797^*^			0.488^*^			0.630^*^
Negative	23 (45.1)	28 (54.9)		16 (48.5)	17 (51.5)		17 (48.6)	18 (51.4)	
Positive	13 (48.1)	14 (51.9)		10 (58.8)	7 (41.2)		10 (55.6)	8 (44.4)	
*GSTP1* ^c^			0.126^**^			0.661^**^			0.420^**^
Negative	34 (49.3)	35 (50.7)		24 (53.3)	21 (46.7)		25 (53.2)	22 (46.8)	
Positive	2 (22.2)	7 (77.8)		2 (40.0)	3 (60.0)		2 (33.3)	4 (66.7)	
*HOXA10* ^c^			0.735^*^			0.704^**^			0.420^**^
Negative	31 (47.0)	35 (53.0)		21 (50.0)	21 (50.0)		25 (53.2)	22 (46.8)	
Positive	5 (41.7)	7 (58.3)		5 (62.5)	3 (37.5)		2 (33.3)	4 (66.7)	
*TP73* ^c^			0.205^**^			0.103^**^			0.192^**^
Negative	34 (48.6)	36 (51.4)		26 (55.3)	21 (44.7)		26 (54.2)	22 (45.8)	
Positive	2 (25.0)	6 (75.0)		0 (0.0)	3 (100.0)		1 (20.0)	4 (80.0)	

TIL: Tumour-infiltrating lymphocytes; EBV: Epstein–Barr virus

a 1: Well differentiated; 2: Moderately differentiated; 3–4: Poorly differentiated and Undifferentiated

b At least one gene

c Methylated status

* Chi-squared test

** Fisher’s exact test
